# Contrast Sensitivity and Colour Vision Tests for Early Detection and Monitoring of Hydroxychloroquine Retinal Toxicity: A Preliminary Study

**DOI:** 10.3390/jcm15031309

**Published:** 2026-02-06

**Authors:** Amal Aldarwesh, Latifah Alwadman, Ali Almustanyir, Mosaad Alhassan, Muhammed S. Alluwimi, Ansam Alateeq, Ibrahim Almaghlouth

**Affiliations:** 1 Optometry Department, College of Applied Medical Sciences, King Saud University, Riyadh 11433, Saudi Arabia; latifahalwdman@gmail.com (L.A.); aalmustanyir@ksu.edu.sa (A.A.); malhassan@ksu.edu.sa (M.A.); ansamalateeq@outlook.com (A.A.); 2 Department of Optometry, College of Applied Medical Sciences, Qassim University, Buraydah 52571, Saudi Arabia; malluwimi@qu.edu.sa; 3 Department of Medicine, College of Medicine, King Saud University, Riyadh 11451, Saudi Arabia; ialmaghlouth@ksu.edu.sa

**Keywords:** hydroxychloroquine, colour vision, contrast sensitivity, autoimmunity

## Abstract

**Background/Objectives**: Hydroxychloroquine (HCQ) is used to manage various autoimmune diseases, including systemic lupus erythematosus. The prolonged use of HCQ is associated with retinopathy and irreversible visual loss due to retinal toxicity. Despite adherence to dosage regimens, patients may develop functional rather than structural changes, without detectable abnormalities on routine examination using visual acuity and optical coherence tomography (OCT). The study aimed to detect early signs of retinopathy in patients with autoimmune diseases treated with HCQ. **Methods**: This cross-sectional study included patients (n = 36) with autoimmune diseases who were treated with HCQ. The control group (n = 35) comprised healthy volunteers matched for age and sex. All participants were screened using colour vision tests (Ishihara, Konan ColourDX high definition [HD]), and retinal thickness was evaluated using OCT. **Results**: Our findings suggest a significant reduction in the contrast threshold of the L and M-cone photoreceptors compared with that of the control using Konan ColourDX HD. The OCT measurements revealed no statistically significant difference in retinal thickness between patients and controls; however, the contrast sensitivity test showed a significant reduction at all spatial frequencies (*p* < 0.0001). **Conclusions**: The current study suggests that the Konan ColourDX cone contrast test HD and contrast sensitivity testing may be valuable for periodic monitoring of patients receiving HCQ, potentially enabling earlier detection of toxicity. However, longitudinal studies with larger cohorts are needed to confirm these findings and to further establish the clinical value of these functional visual tests.

## 1. Introduction

Hydroxychloroquine (HCQ) is an aminoquinoline derivative that was introduced as an antimalarial drug and was later used as an immunosuppressant for managing systemic lupus erythematosus (SLE) in the early 1950s because of its anti-inflammatory and immunomodulatory effects [1,2]. The proposed mechanisms underlying these effects include the reduction in pro-inflammatory cytokines, including tumour necrosis factor and interleukins 1 and 6. Additionally, HCQ blocks the activation of toll-like receptors in immune cells, further reducing the production of pro-inflammatory cytokines. Moreover, HCQ has an antithrombotic effect stemming from the inhibition of phospholipase A2 activity, which decreases platelet aggregation [1,2]. Therefore, the use of HCQ has been extended to other rheumatological disorders, including rheumatoid arthritis (RA), primary Sjögren syndrome and antiphospholipid syndrome [3].

Despite its safety profile and effectiveness in controlling disease activity, retinal toxicity remains a bothersome complication in patients, particularly those who require increasing doses of the drug or have a longer treatment duration. A prevalence of HCQ retinal toxicity ranging from 0.08% to 30% across different populations was observed [4,5,6]. In Saudi Arabia, a prevalence of 1.58% was reported by Adel et al. (2020) over an average 8-year treatment period with a higher dose of HCQ [7].

Recent recommendations from the American Academy of Ophthalmology (AAO) indicate that the risk of retinal toxicity increases in the following categories: patients receiving doses >5 mg/kg/day real weight, duration of use >5 years, presence of renal disease, concomitant use of tamoxifen, and underlying retinal and macular disease [8].

The retinopathy described in clinical research suggests that HCQ could cause the following patterns of damage: peripheral extramacular, parafoveal, pericentral and mixed patterns [3,9,10,11,12].

A more severe clinical picture of HCQ toxicity was classically described as bilateral bull’s eye maculopathy, which has been observed in the past with a high dosage of 6.5 mg/kg ideal weight. The mechanism of HCQ-induced retinal toxicity is not fully understood; however, data from experimental studies suggest that HCQ binds to melanin in the retinal pigment epithelium (RPE) and accumulates in tissues, undermining the protective role of melanin as a free radical scavenger [13]. Damaged RPE cells migrate to the outer and inner retinas, resulting in irreversible photoreceptor loss [14]. If damage is detected before RPE loss, central vision can be preserved. However, if bull’s eye manifests, progressive foveal thinning and loss of visual acuity are imminent.

In the early stages of treatment, HCQ-induced photoreceptor toxicity is unlikely, particularly in well-controlled patients who do not receive an appropriate cumulative dose [15]. Other studies have indicated that patients treated with HCQ have lower cone density in the vertical axis and inferior, nasal, and temporal quadrants, with a larger cone spacing in these areas [16,17]. Cone loss increases as cumulative doses of HCQ increase, despite no clinical evidence of maculopathy during routine screening tests using optical coherence tomography (OCT) [18].

HCQ toxicity is detected using visual field testing, spectral-domain optical coherence tomography (SD-OCT), and multifocal electroretinography; however, AAO renders colour vision testing insensitive and non-specific [6]. According to AAO recommendations, all patients prescribed HCQ should have a baseline ophthalmologic examination during the first year of treatment to assess their functional status. Automated visual field (VF) testing and SD-OCT are both recommended for primary screening, with the latter considered more specific and sensitive for detecting damage, whereas VF testing varies between visits and requires reliability on the part of patients [8,19]. Moreover, SD-OCT detects HCQ-induced photoreceptor damage in the parafoveal region, such as localized thinning [8]. Fundus autofluorescence (FAF) provides an additional topographic view of the RPE damage and early damage to the parafoveal area. In addition, multifocal electroretinography (mfERG) objectively detects early retinopathy in the form of parafoveal and extramacular depression [8,19]. In 2025, the AAO guidelines prioritized OCT, along with wide-pattern fundus autofluorescence, while VF and mfERG are considered secondary tests [20]. On the other hand, OCT angiography, time-domain OCT, fluorescein angiography, full-field electroretinogram (ERG), and colour vision tests remain excluded [20,21,22,23].

However, studies have reported that contrast sensitivity and colour vision tests can be used to detect signs of early visual dysfunction in HCQ users, who may experience a reduction in central and peripheral contrast sensitivity [24,25], marked changes in tritan colour vision [26] and reduced cone densities [16,17,18].

In the current study, we aimed to use colour vision and contrast sensitivity tests to assess the visual function in HCQ users to detect early changes in the function of photoreceptors and assist in clinical decision-making in patients who require continuous monitoring, prognose those who may potentially progress to retinopathy, or decide upon discontinuation of the drug in patients with risk factors.

## 2. Materials and Methods

### 2.1. Research Participants and Ethical Considerations

This cross-sectional study involving patients with autoimmunity (n = 36) was conducted at the Rheumatology Clinic of King Khalid University Hospital. Patients were recruited during regular follow-up appointments with a rheumatologist, and informed consent was obtained before participation. Control participants (n = 35) were recruited from outpatient clinics and visitors in waiting areas and invited to participate in the research following a short interview to confirm that they were free of autoimmune diseases and chronic conditions, including diabetes and glaucoma. Medical history data of patients with autoimmunity were obtained from their medical records. The research protocol was approved by the Institutional Review Board subcommittee of the College of Medicine at King Saud University (approval no. E–20–530). Informed consent was obtained from all participants before data collection and ocular examination. The study was conducted in accordance with the Declaration of Helsinki.

### 2.2. Ocular Examination

First, visual acuity was assessed using the Snellen visual acuity chart, followed by a contrast sensitivity test using the contrast sensitivity vision (CSV)-1000e chart, colour vision tests which include the Ishihara, Hardy–Rand–Rittler (HRR), and ColourDX cone contrast test (CCT) HD, and lastly, OCT was used.

### 2.3. Visual Acuity

The visual acuity test was performed using a tumbling E-chart at 6 m under monocular vision. All participants were corrected based on objective refraction to ensure that the reduction in visual acuity was associated with retinopathy due to drug use and was unrelated to other causes, such as uncorrected refractive error. All participants had a best-corrected VA of 6/6 with a LogMAR value of 0.0. There were no significant differences between the two groups.

### 2.4. Contrast Sensitivity

The contrast sensitivity was tested using the CSV-1000e (Good-Lite, Elgin, IL, USA). at a distance of 2.5 m with a movable screen and under photopic conditions, which represent daylight vision dominated by cones. The CSV-1000e provides four rows of sine-wave gratings with randomized locations for the grating targets. The gratings were tested for spatial frequency (SF) at 3, 6, 12, and 18 cycles per degree. The CSV test provides a contrast sensitivity function (CSF) curve. The contrast sensitivity values for each SF were plotted against the specified SF, resulting in a graph displaying the CSF.

### 2.5. Colour Vision Tests

#### Ishihara and HRR Colour Vision Tests

The Ishihara test, 38 plate edition (Kanehara Trading Inc., 2011; Tokyo, Japan), is a screening tool used to assess red–green congenital colour deficiency by presenting plates with numbers or patterns formed by coloured dots of varying sizes and intensities set against a background of differently coloured dots. Under a standard light source simulating natural daylight, the patient, using their near vision correction and holding the booklet at 75 cm, was asked to read the numbers starting with plate one, with each plate shown for no more than 3 s. Some plates are visible only to individuals with normal colour vision, whereas others are designed to be observed by those with red–green colour vision defects. The assessment focuses on the reading of plates 1 to 21 to determine normal or defective colour vision, with a passing score of 17 or more plates read correctly indicating normal colour vision, whereas more than three errors in the entire test were considered failures. On the other hand, the HRR plates 4th edition (Richmond Products, Albuquerque, NM, USA) assesses red–green and blue–yellow colour vision deficiencies. The test comprised 24 plates representing simple shapes, such as circles and triangles. HRR includes four demonstration plates, six for screening, and 14 for diagnosing colour deficiency. A passing score was obtained when the participants correctly identified all six screening platforms, and the results were recorded on a sheet to keep track of errors during the test.

### 2.6. OCT Measurements

The OCT (Topcon 3D OCT-1 Maestro, Topcon Corporation, Tokyo, Japan) is a non-invasive imaging test that uses light waves to take cross-sectional scans of the retina and is used to measure the outer retinal layer thickness. The examination protocol used was 3D macular (V) volume. The macula was examined using 512 A × 128 B scans covering an area of 7 × 7 mm centred on the fovea and divided into 10 × 10 grids. The outer retinal thickness was obtained by subtracting the thickness of the inner retinal layer from the total thickness.

### 2.7. Konan ColourDX CCT HD

The Konan ColourDX HD (Konan Medical, Irvine, CA, USA) is a high-precision diagnostic colour vision test that uses a computerized cone contrast threshold method with a Landolt C optotype randomly presented to the participant in one of four directions. The test began by adjusting the eye of the subject to the centre of the screen, occluding one eye and ensuring that they wore glasses if the participant had presbyopia. The participants sat 60 cm away from the monitor in a dimly lit room. The screen briefly shows a ‘C’ shape in various colours and directions, and the participant uses the arrow keys to indicate the direction of the opening within 2–3 s. A high tone indicates a correct response, whereas a low tone indicates an error. Because the test measures thresholds below normal visual ability, guessing is encouraged if a gap is not observed. The test targets individual cone types: L-cone (long wavelength), M-cone (medium wavelength), and S-cone (short wavelength) by stimulating them through an opponent mechanism, providing a quantitative threshold and logarithm of contrast sensitivity for each cone type. The results were displayed and automatically saved in a database [27].

### 2.8. Statistical Analysis

Data were analysed using Microsoft Excel (V.15.0) and GraphPad Prism (version 8.4.3, for Mac, GraphPad Software, La Jolla, CA, USA, www.graphpad.com). Normality was assessed using the Shapiro–Wilk test and Q–Q plots. Based on the normality results, the measured variables were compared between the two groups using either an unpaired *t*-test or Mann–Whitney. *p*-values < 0.05 were considered statistically significant.

## 3. Results

### 3.1. Research Participants

The study included 36 autoimmune disease patients with an average age of 35.5 ± 11.6 years, of whom 97% were females treated with HCQ at the time of recruitment. Their data were compared with those of healthy controls matched for age, sex, and visual acuity (Table 1). All participants showed normal colour discrimination on the Ishihara and HHR tests.

Most patients (77%) had SLE without ocular or colour vision defects. Hypertension and diabetes mellitus were reported in four patients (11.11%) in the study group. Secondary autoimmunity, including hypothyroidism, was present in eight patients, whereas Sjogren’s syndrome affected two patients as a secondary condition. The duration of the autoimmunity ranged from 1 to 20 years, with an average of 8.4 ± 5.6 years (Table 2). Most patients had active inflammation, as evidenced by elevated C-reactive protein and erythrocyte sedimentation rate levels.

### 3.2. HCQ Treatment

The body weight of patients in the study group ranges from 39 to 116 kg with an average of 69.9 ± 16.4 kg. However, most patients (66.7%) were maintained on a daily HCQ tablet equivalent to 200 mg, regardless of their body weight (Table 3). Treatment with HCQ exceeded the five-year duration for most patients with SLE (42%), a minor proportion of patients (22%) exceeded 10 years of treatment, and the remaining patients received the drug for less than 5 years. Patients received a cumulative dose of HCQ that ranged from 73 to 2482 g, exceeding the cut-off point of 1000 g in seven patients (19.4%), while the majority (50%) remained within the safe range of 0 to <500 g (Table 3).

### 3.3. Retinal Thickness Examination

A retinal scan using Topcon 3D OCT-1 of the total retinal layer thickness (Table 4), inner retinal layer thickness (Table 5), and outer retinal layer thickness (Table 6) showed no significant differences in the thickness of all quadrants compared to healthy individuals without autoimmunity.

### 3.4. Effect of HCQ on the Contrast Threshold of the Cone Photoreceptors and Contrast Sensitivity

**Figure 1:**
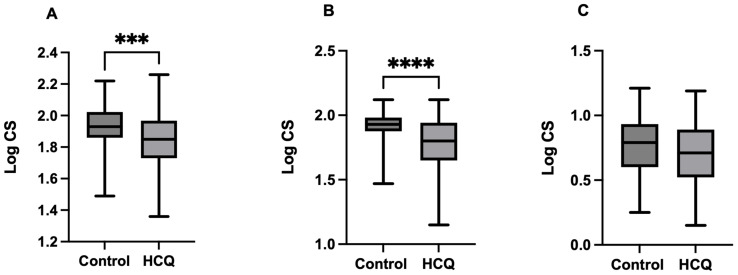
Effect of hydroxychloroquine (HCQ) on (**A**) L-cone, (**B**) M-cone, and (**C**) S-cone. Statistical significance was determined as follows: *** *p* < 0.001, **** *p* < 0.0001.

Data obtained by the ColourDX CCT HD test indicate a significant reduction in the Log CS of L-cones (mean difference = −0.103 ± 0.03, t = 3.66, df = 140, *p* = 0.0004) and M-cones (Mann–Whitney U = −2092, *p* < 0.002) while the Log CS of the S-cone shows non-significant levels between the two groups (mean difference = −0.07 ± 0.04, t = 1.94, df = 140, *p* = 0.054) (Figure 1).

### 3.5. Effect of HCQ on Contrast Sensitivity

**Figure 2:**
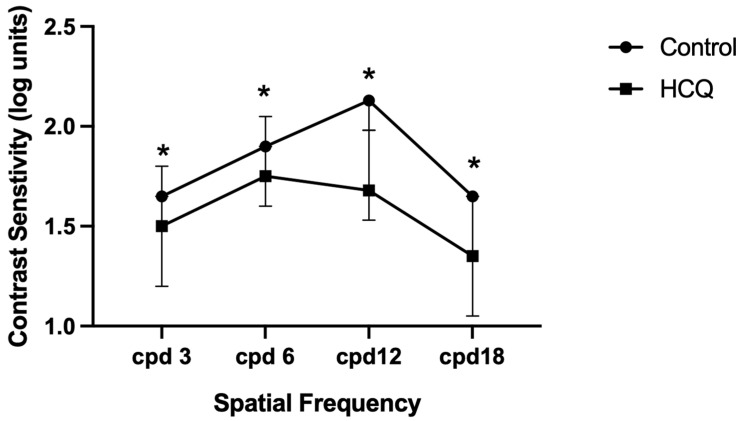
Contrast sensitivity function in patients taking HCQ and controls. Contrast sensitivity is shown as log units across four spatial frequencies for HCQ-treated patients and controls. Data are presented as medians, with error bars representing the interquartile range (Q1–Q3). * Differences between groups were statistically significant at all spatial frequencies (*p* < 0.000).

The log units of contrast sensitivity at four SFs were compared between HCQ-treated patients and controls. Data are expressed as medians with first and third quartiles (Q1–Q3) and 95% confidence intervals (Table 7). Contrast sensitivity was significantly lower in HCQ-treated patients at all SFs (*p* < 0.0001). Figure 2 shows the median log units for each SF, with error bars representing the interquartile range (IQR).

## 4. Discussion

HCQ is a hydroxylated derivative of chloroquine initially designed to treat malaria and extraintestinal amoebiasis. HCQ is currently prescribed for the management of autoimmune inflammatory conditions, including RA and SLE, either alone or in combination with other immunomodulatory agents [28]. The ocular toxicity of HCQ is dose- and time-dependent; patients who receive higher cumulative doses over many years or even decades have a higher risk of developing bull’s eye maculopathy, characterized by central vision loss, reading difficulty, and reduced colour perception [13,26]. In the current study, we examined patients with autoimmune diseases, mainly SLE, with a variable duration of HCQ use (between 1 and 20 years) who received an appropriate dosage regimen. Retinal examination of patients with autoimmunity using OCT revealed no structural abnormalities or changes in the total, outer, and inner retinal thicknesses compared to healthy individuals. In contrast, many studies have reported manifestations of retinal toxicity detected using OCT with a higher duration or dosage of HCQ. In Indian patients with autoimmune diseases, HCQ toxicity manifests as a parafoveal or a combination of parafoveal and perifoveal patterns with a cumulative dose of more than 1000 and a daily dose of more than 5 mg/kg [28]. Thinning of the parafoveal and perifoveal regions was reported in 10% of a cohort of Indian patients with rheumatic diseases, in which three patients were maintained at a mean daily dose of <5 mg/kg/day. Toxicity was confirmed in approximately 45% of the patients using spectral-domain optical coherence tomography (SD-OCT), whereas the rest had abnormalities confirmed through the automated visual field [29]. Similar to our demographic characteristics, Mondal et al. [14] examined 19 patients with a history of chronic HCQ use to treat RA and SLE, with no ocular pathology, and a duration of 1–20 years. The median HCQ use was less than 5 years, and the median daily dose was 4.65 mg/kg. Retinal SD-OCT images revealed retinal toxicity in five patients in the form of central macular thickness reduction, central cube volume, and the thickness of the combined ganglion cell and inner plexiform layer.

HCQ reduces colour perception, preferentially affecting S-cones more than L- and M-cones [13,26,30,31]. Consistent with these clinical findings, animal studies have shown that chronic HCQ exposure affects both inner and outer retinal neurons, leading to a significant reduction in ganglion cell–inner plexiform layer thickness and retinal ganglion cell numbers compared with controls, in a pattern similar to retinal toxicity observed in humans [14]. Furthermore, chronic HCQ exposure in mice resulted in a significant reduction in visual acuity that progressively worsened over a 3-month treatment period and was accompanied by impaired retinal rod and cone function. However, contrast sensitivity was not affected [14]. Photoreceptors may be affected at different stages of HCQ treatment without altering the retinal layer thickness, as indicated by our results, which showed a significant reduction in the thresholds of the L- and M-cones. This contrasts with previous reports [30,31] that indicated that S cones are more affected by HCQ toxicity, whereas our study revealed a variable response in S-cone photoreceptors. Based on the fragile receptor hypothesis, S-cones, which are present in lower numbers than L- and M-cones, are more susceptible to damage in retinal diseases such as diabetic retinopathy and retinitis pigmentosa. This is attributed to their high metabolic demands, which make them vulnerable to stress and prone to loss earlier than L- and M-cones [32,33].

Studies have reported that acquired colour deficiency is an early indicator of drug-induced retinopathy, manifested as a tritan defect (blue–yellow), indicating the reduced sensitivity of S-cones [34,35]. In addition, patients with confirmed HCQ-induced retinopathy and typical bull’s eye maculopathy showed an abnormal yellow–blue colour threshold [26,36]. Since our patients had no evidence of retinal toxicity, our data may be explained by early cone dysfunction rather than selective cone damage and loss [37]. HCQ retinopathy most commonly affects the parafoveal region, which contains a mixed population of L- and M-cones, with few S-cones. Hence, the reduction in contrast sensitivity may be dominated by L/M-cone input. The non-significant reduction in the log CS of S-cones observed in our study may reflect the test sensitivity and sample size rather than the true absence of short wavelength pathway involvement. Several studies have shown that HCQ treatment alters the cone density despite a normal retinal structure and may contribute to reduced contrast sensitivity. In a study that involved 23 SLE patients treated with HCQ with no evidence of retinopathy, who received a cumulative dose of 2–2160 g, there was a negative correlation between the parafoveal cone density and the HCQ cumulative dose [18].

A study by Tang et al. involving patients with rheumatic diseases, with a total cumulative hydroxychloroquine dose of 368.3 ± 267.2 g and a HCQ treatment duration of 36.5 ± 26.5 months, showed a significant reduction in cone density in the vertical axis in the patients, as compared with healthy controls [16]. In a more recent study involving 18 women with autoimmunity who exceeded a cumulative dose of 1600 g and had no evidence of toxicity as assessed via OCT, visual field, and multifocal electroretinography, the patients’ cone density was significantly lower than in the controls [17].

Our findings also indicated that the contrast sensitivity measurements were significantly reduced in HCQ users at all SFs of 3, 6, 8, and 18 cycles/degree. This finding is in agreement with a recent report by Sonalcan et al. [25], who observed that contrast sensitivity measurements were significantly lower in the HCQ group than in the control group at all SFs, except 6 and 18 cycles/degree [19]. A previous study by Singla et al. [24] suggested that HCQ affects contrast sensitivity by acting on bipolar and ganglion cells. This effect can be detected as early as 3 and 6 months post-HCQ treatment and could be considered an early sign of visual dysfunction. The observed functional changes in contrast sensitivity and cone thresholds may be attributed to a potentially reversible pharmacological effect on photoreceptor metabolism, considering that patients with low and high cumulative doses had a normal OCT result. However, to support this claim, we need a longitudinal study of patients free from the risk factors of toxicity who continue to receive HCQ. On the other hand, evidence from clinical and experimental studies suggests that HCQ could compromise visual function at the early stages of exposure. Importantly, visual acuity and contrast sensitivity were reduced in HCQ-treated mice after one month of exposure [14].

Data from in vitro studies revealed that chloroquine inhibits the phagocytosis of the rod photoreceptor outer segment [38]. Moreover, HCQ inhibits the uptake of all-trans-retinol, which disturbs the visual cycle [39]. The binding of HCQ to melanin in the pigment epithelium impairs its antioxidant function, enabling drug accumulation and eventually leading to RPE degeneration [19].

In vitro and in vivo studies have shown that the exposure of photoreceptors to HCQ inhibits autophagy in a dose-dependent manner, leading to alterations in sphingolipid metabolism [40]. Moreover, chronic HCQ treatment is toxic to the inner retinal neurons, and this manifests before the development of bull’s eye maculopathy and RPE atrophy [14].

In humans, HCQ-induced retinal toxicity has been reported to occur in some patients despite adherence to the appropriate dosage. In a cohort study by Almeida et al., 1460 patients were followed from 1999 to 2019. Retinal toxicity was confirmed in 11 patients, and the lowest HCQ was 3.6 mg/kg/day, while 3 patients were free from risk factors of toxicity [41].

In advanced cases of HCQ toxicity characterized by bull’s eye maculopathy, outer retinal toxicity is evident with macular mottling. Further retinal damage involves the progressive and irreversible atrophy of the retinal pigment epithelium despite discontinuation of the drug [42].

This study had several limitations, including a small sample size, due to the restricted inclusion criteria aimed at avoiding multiple comorbidities, such as progressive cases of autoimmunity that necessitate systemic medications. In addition, the lack of an HCQ-naive autoimmune control group remains a constraint. Patients were recruited using a cross-sectional design during clinic visits; therefore, they had varying disease durations and HCQ dosages, resulting in a wide range of cumulative doses. Moreover, it was not feasible to investigate patients’ adherence to HCQ and the timing of dosage changes or discontinuation, if any, during the disease course. Although most patients had SLE, a small proportion of participants had RA, and SS may have introduced heterogeneity in disease mechanism and ocular involvement. Identifying a more homogeneous group from medical records and subsequently inviting them to participate was not feasible, as the study timeframe did not permit waiting 6 months or longer for patients to attend scheduled appointments at the university hospital, and compliance could not be guaranteed.

## 5. Conclusions

The data derived from this study are preliminary and do not differentiate between early and potentially reversible photoreceptor toxicity associated with HCQ treatment and established and irreversible retinal damage. Nevertheless, the findings are promising and suggest that early HCQ-related changes may be detectable using ocular assessments such as colour vision and contrast sensitivity testing, warranting further longitudinal investigation. These modalities may assist clinicians in monitoring and predicting outcomes in patients treated with HCQ at different stages of disease, particularly in the early clinically silent phase when toxicity is not apparent on routine examination. Further longitudinal studies with larger cohorts and more homogeneous cohorts, with appropriate adjustment for potential confounders, are required to confirm these findings using functional visual tests.

## Figures and Tables

**Table 1:** Demographic data of participants.

		Normal	Autoimmune Disease	Test	*p*-Value
Sample Size (n)		35	36		
Age (years)	Mean ± SD	35.3 ± 9.2	35.2 ± 10.0	t = 0.064 df = 68.82	0.95
Sex n (%)	Males	---	1 (2.78%)		
	Females	35 (100%)	35 (97.2%)		
Visual Acuity Median LogMAR (IQR)	OD	0.00 (0.00–0.00)	0.00 (0.00–0.00)	U = 562	0.2
	OS	0.00 (0.00–0.00)	0.00 0.00–0.00	U = 539.5	0.07

Abbreviations: dfs, degrees of freedom; IQR, interquartile range; OD, right eye; OS, left eye; t, *t*-test statistic; U, Mann–Whitney U statistic.

**Table 2:** Characteristics of patients treated with hydroxychloroquine.

Parameter	Value	
Medical History		
Primary autoimmune disease, n (%)	SLE	28 (77.1%)
	RA	7 (19.4%)
	SS	1 (2.8%)
Secondary autoimmune disease, n (%)	HT	8 (22.2%)
	SS	3 (8.3%)
Presence of chronic conditions, n (%)	HTN	3 (8.3%)
	DM	1 (2.8%)
Duration of primary autoimmunity (years)	Median	8
	Q1–Q3	3.5–12
	Range	1–20
Presence of ocular disease, n (%)	0.0	
Presence of colour vision defects, n (%)	0.0	
Levels of Inflammatory Markers		
ESR (mm/hr)	Mean ± SD	32.5 ± 19.3
	Range	2–72
CRP (mg/L)	Mean ± SD	6.5 ± 10.1
	Range	0.21–46.9

Abbreviations: SLE, systemic lupus erythematosus; RA, rheumatoid arthritis; SS, Sjogren’s syndrome; HT, hypothyroidism; HTN, hypertension; DM, diabetes mellitus; ESR, erythrocyte sedimentation rate; CRP, C-reactive protein; Q, quartile.

**Table 3:** Characteristics of hydroxychloroquine (HCQ) treatment among patients with autoimmune diseases.

Parameter	Value	
Body weight (Kg)	Mean ± SD	69.9 ± 16.4
	Range	39–116
Current dose of HCQ (200 mg tablet)	One	25 (69.4%)
	Two	11 (30.6%)
	
Daily dose (mg/Kg/day)	Mean ± SD	3.6 ± 1.3
	Median	3.7
	Range	2.2–7.4
	<4.0	23
	4.0–5.0	8.0
	>5.0	5.0
Duration for HCQ treatment (years)	Mean ± SD	6.8 ± 5.3
	Range	1.0–19
	<5.0	13
	5.0–10	15
	>10	8
Predicted cumulative (g)	Median	438
	Range	73–2482
	0–<500 g	19
	500–1000 g	10
	>1000	7.0

**Table 4:** Total retinal layer thickness as determined using Topcon 3D optical coherence tomography (OCT)-1 Maestro.

Parameter (mm)	Normal (n = 35)	HCQ (n = 36)	*p*
Right eye			
Retinal quadrants average	274.8 ± 13.3	278.3 ± 12.2	0.27
Superior retina	276.7 ± 18.3	279.6 ± 17.9	0.37
Inferior retina	272.9 ± 17.6	276.9 ± 15.6	0.18
Left eye			
Retinal quadrants average	284.3 ± 13.4	278.9 ± 12.8	0.39
Superior retina	278.1 ± 18.2	280.4 ± 17.1	0.48
Inferior retina	274.5 ± 16.5	277.5 ± 16.5	0.33

**Table 5:** Inner retinal layer thickness as determined using Topcon 3D OCT-1 Maestro.

Parameter (μm)	Normal (n = 35)	HCQ (n = 36)	*p*
Right eye			
GCL++ average	107.4 ± 7.2	109.8 ± 6.6	0.16
GCL++ superior	106.8 ± 12.1	109.1 ± 12.2	0.27
GCL++ inferior	108.03 ± 11.4	110.4 ± 10.5	0.20
Left eye			
GCL++ average	108.5 ± 7.2	109.9 ± 7.6	0.42
GCL++ superior	107.8 ± 11.9	109.6 ± 11.7	0.44
GCL++ inferior	109.3 ± 11.6	110.6 ± 12.9	0.16

Abbreviations: GCL++, ganglion cell layer ++: a combination of retinal nerve fiber layer, RNFL; ganglion cell, GCL; and inner plexiform layer, IPL).

**Table 6:** Outer retinal layer thickness as determined using Topcon 3D OCT-1 Maestro.

Parameter (μm)	Normal (n = 36)	HCQ (n = 35)	*p*
Outer retina—right eye			
Superior nasal	170.6 ± 12.5	172.5 ± 10.5	0.50
Superior temporal	165.1 ± 13.7	168.8 ± 9.9	0.22
Inferior nasal	165.5 ± 13.3	167.8 ± 10.1	0.41
Inferior temporal	160.5 ± 13.9	165.2 ± 8.9	0.10
Quadrants average	165.4 ± 13.1	168.5 ± 9.3	0.26
Outer retina—left eye			
Superior nasal	168.9 ± 10.6	169.4 ± 9.2	0.83
Superior temporal	170.8 ± 10.4	172.6 ± 10.6	0.47
Inferior nasal	164.2 ± 8.7	166.4 ± 9.4	0.32
Inferior temporal	165.3 ± 9.3	167.5 ± 10.3	0.36
Quadrants average	167.3 ± 9.2	168.9 ± 9.5	0.46

**Table 7:** Contrast sensitivity tests.

	Normal (n = 35)				HCQ Group (n = 36)					
Contrast Sensitivity	Median	95% CI	Q1	Q3	Median	95% CI	Q1	Q3	U	***p***-Value
SF 3 cpd	1.65	1.62–1.72	1.65	1.80	1.50	1.33–1.52	1.20	1.65	289.5	<0.000
SF 6 cpd	1.90	1.89–1.98	1.90	2.10	1.75	1.69–1.85	1.60	1.90	348	0.0001
SF 12 cpd	2.13	1.98–2.07	1.98	2.13	1.68	1.64–1.84	1.53	1.98	280.5	<0.000
SF 18 cpd	1.65	1.58–1.64	1.65	1.65	1.35	1.16–1.41	1.10	1.65	300.5	<0.000

Abbreviations: 95% CI: 95% confidence interval; cpd: cycle per degree; Q1: first quartile (25%); Q3: third quartile (75%); SF: spatial frequency; U: Mann–Whitney U test.

## Data Availability

The original contributions presented in this study are included in the article. Further inquiries can be directed to the corresponding author.

## Abbreviations

The following abbreviations are used in this manuscript:

HCQ	Hydroxychloroquine
OCT	Optical coherence tomography
SLE	Systemic lupus erythematosus
RA	Rheumatoid arthritis
AAO	American Academy of Ophthalmology
RPE	Retinal pigment epithelium
HD	High definition
CSV	Contrast sensitivity vision
CSF	Contrast sensitivity function
SF	Spatial frequency
HRR	Hardy–Rand–Rittler
CCT	Cone contrast test
GCL	Ganglion cell layer
CI	Confidence interval
SD-OCT	Spectral-domain optical coherence tomography
